# Using** Concept Mapping** to** Explore the Perspectives of People with Mild to Borderline Intellectual Disabilities** **Toward Sexual Health**

**DOI:** 10.1007/s11195-023-09796-w

**Published:** 2023-05-09

**Authors:** Wouter de Wit, Diana Roeg, Petri J. C. M. Embregts

**Affiliations:** 1 Tilburg School of Social and Behavioral Sciences, Tilburg University, Postbus 90153, 5000 LE Tilburg, The Netherlands; 2 Zuidwester, Middelharnis, The Netherlands; 3 Kwintes Supported Housing, Zeist, The Netherlands

**Keywords:** Intellectual disabilities, Support staff, Relatives, Sexual health, Concept mapping, The Netherlands

## Abstract

People with mild to borderline intellectual disabilities face many barriers toward their sexual health. To promote sexual health and overcome these barriers, they need individualized forms of sexuality support and education. To align sexuality support and education insight is needed on their understanding of sexual health. The current paper aims to provide greater insight in what sexual health consists of according to people with mild to borderline intellectual disabilities. Nine people with a mild to borderline intellectual disability participated in a concept mapping procedure, consisting of brainstorming, sorting, and ranking the statements. The resulting clusters and concept map were interpreted by an expert group. Analysis resulted in five clusters which participants considered important for their sexual health. According to people with mild to borderline intellectual disability cluster pertaining to romantic relationships and sexual socialization were the most important. These were followed by clusters on sexual health and lastly sexual selfhood. These results have implications for the development of sexuality support and education, as well as further research.

**Supplementary Information:**

The online version contains supplementary material available at 10.1007/s11195-023-09796-w.

## Introduction

Sexual health can be defined as ‘a state of physical, emotional, mental and social well-being in relation to sexuality’, and, most notably, consists of having sexual relationships and being able to engage in pleasurable and safe sexual experiences [1]. To develop sexual health has also been found to be necessary for meaningful, long-term romantic relationships [2]. Although recent studies have consistently shown that people with mild to borderline intellectual disabilities (ID) have the same sexual needs and desires as people without ID [3], people with mild to borderline ID have less positive sexual experiences [4, 5]. While most people with mild to borderline ID have experience with hugging and kissing, vaginal intercourse is less common among this group, with most estimates ranging between 51 and 84.4% [6–8]. In comparison, somewhere around 95% of adults without ID are estimated to engage in vaginal intercourse [7, 8]. Furthermore, people with mild to borderline ID may encounter higher sexual risks, insofar as 32.9% of this group are estimated to have experienced sexual abuse, in comparison to 8.9% of adults without ID [9]. Promoting sexual health in people with mild to borderline ID is crucial to enable them to enjoy fulfilling relationships, reduce the risk of sexual abuse, and lead satisfying lives [10, 11]. However, the specific strategies for achieving this goal remain unclear [12]. To advance these strategies, the present study focusses on the perspectives of people with mild to borderline ID on their sexual health.

People with mild to borderline ID face many barriers related to their sexual health [4, 13], including, among other things, social stigma, both at a societal level and more directly from support staff and relatives [4, 12, 14]. For many years, the general attitude toward people with mild to borderline ID has been that they are asexual or have a childlike sexuality, with the implication being that they are incapable of engaging in reciprocal sexual relations [2, 15]. Hence, support staff and relatives often perceive sexual expressions as acts of friendship or self-exploratory behavior [16–18]. Alongside this, support staff and relatives may perceive people with mild to borderline ID as sexually vulnerable, either as a victim or a perpetrator of sexual abuse [4, 19, 20]. As a result of these stereotypes, support staff and relatives have been found to either ignore or limit the sexuality of people with mild to borderline ID [7, 19].

People with mild to borderline ID also experience barriers in their sexual health at an individual level, namely in terms of their cognitive and social functioning [4, 21]. For example, some people with mild to borderline ID find it difficult to meet potential partners [22, 23], insofar as they perceive their disability to be a barrier in dating, do not know how to date, or feel embarrassed when attempting to date [24, 25]. For some, meeting potential partners is difficult, because they live largely segregated lives either within institutions or with their parents [26]. Given that they receive support, some people with mild to borderline ID report being reluctant to explore their sexual health out of fear of being restricted and punished by support staff and relatives [21, 27]. When viewed together, these aforementioned barriers stemming from support staff, relatives and people with mild to borderline ID themselves, serve to undermine their ability to engage in sexual experiences as well as receive the support and education that is necessary for them to develop their sexual health [7, 20].

To overcome these barriers toward sexual health, it is important that people with mild to borderline ID have access to individualized forms of support and education [28, 29]. However, their sexual health has long been neglected in research and practice due to social stigma and paternalism [30], with most studies focussing on the perspectives of support staff and relatives rather than people with mild to borderline ID themselves [31]. Consequently, these results may fail to meet the needs of people with mild to borderline ID in support and education [29], with problematic effects on their sexual health [32]. To ensure future support and education aligning the needs of individuals with mild to borderline ID, it is necessary to gain further insights into their perspectives toward sexual health [5, 33]. The present study aims to fill this gap in knowledge by exploring their perceptions of sexual health, providing valuable information to inform future support and education.

## Method

### Design

This paper reports on the perspectives of people with mild to borderline ID toward their sexual health. The method of concept mapping was utilized, because it allows for the integration of a complex concept into a visual representation through the combination of qualitative and quantitative methods [34]. This method structures the process through which a group perspective is formed, while, simultaneously, maintaining the individual perspectives [34]. This method has been applied successfully in various healthcare studies [35], including studies on sexuality [36] and within the field of ID [37, 38]. Ethical approval was granted by the Ethics Review Board [name of university removed for the purposes of blind peer review].

### Participants

The participants were recruited through purposive sampling from a healthcare organization for people with ID in the Netherlands. Predefined selection criteria were set based on gender and age, in order to ensure that a wide variety of opinions were included and enhance the validity [34]. Specifically, eligible participants had to be men and women aged over 18 years, who had a mild to borderline ID (IQ-score between 50 and 85). The > 18 years criterion was used because sexuality prior to the age of 18 is subject to many physical and hormonal-level changes [39]. Our study had no prerequisites for participation related to sexual experience, gender identity, or sexual orientation. As people with mild to borderline ID are at higher risk for psychiatric and physical comorbidity [40, 41], which is associated with unique sexual needs [42], there were no exclusion criteria set for comorbidity in order to enhance the validity of the study. Independent healthcare psychologists from the healthcare organization asked eligible candidates’ permission to share their contact information for the purpose of arranging an informative meeting with the researchers. The first author made the initial contact by phone and invited eligible candidates to attend a one-on-one introductory meeting where further information was provided. In total, nine people with mild to borderline ID agreed to a meeting and were informed by the first author about the nature of the study via a standardized information letter. Three participants, including two women, used the offer to invite a staff member along for support during the information meeting. After these meetings, the seven men and two women provided their written informed consent to participate in the present study. The characteristics of the participants are presented in Table 1.

**Table 1:** Participants’ demographics

Gender^a^	M	M	M	M	M	M	M	F	F
Age	31	59	58	29	42	48	36	22	28
Level of ID	Borderline	Mild	Mild	Mild	Borderline	Mild	Mild	Mild	Mild
Chronic medical diagnosis	–	–	Tetraplegia	–	–	Epilepsy	Hemiplegia	–	–
Psychiatric diagnosis	Autism spectrum disorder, schizophrenia, alcohol abuse in remission	–	–	–	–	–	–	–	Borderline personality disorder, posttraumatic stress disorder
Living arrangements	Residential group home	Residential group home	Residential group home	Community supported living (own apartment)	Residential group home	Residential group home	Community supported living (own apartment)	Residential group home	Residential group home

^a^With respect to gender: ‘M’ refers to men, and ‘F’ refers to women

### Procedure

The concept map procedure consists of four consecutive steps [34]. In the first step, a brainstorming session was held in two online focus groups. The first group consisted of seven men with mild to borderline ID. To include the female perspective, a second focus group was held with two women with mild ID. The focus groups were facilitated by a male and female researcher, both of whom had experience interviewing people with mild to borderline ID and conducted over Microsoft Teams. All the focus group sessions were video recorded.

After a brief introduction explaining the purpose and background of the research, the participants were asked to answer the following focal question: ‘*Sexual health for people with mild ID consists of …*.’ The participants were encouraged to state as many ideas as possible. When appropriate, the researchers asked for clarification to facilitate the discussion and brainstorming of ideas. All the statements were noted live on-screen to facilitate feedback. The brainstorming finished when data saturation was achieved. In the first group, data saturation was achieved after approximately 50 min, while in the second group it occurred after 20 min, due to the smaller number of participants. Afterwards, the resulting list of statements was checked by the researchers to ensure clarity, remove duplicates, merge overlapping ideas, and remove any statements that did not answer the focal question.

In the second step, the participants structured the resulting ideas via an individual sorting and ranking task. They received practical assistance from the first author, and if the participants encountered any difficult words the researcher clarified the meaning for them upon request. Prior to commencing the sorting task, all statements were read aloud by the first author. Next, the statements were handed out on printed cards one at a time, while being read aloud again. After receiving each card, the participants single-handedly sorted the cards into piles which they considered to be of similar meaning. Once a pile consisted of five statements, the participants were asked to label the pile. At this point, the label primarily functioned as a ‘placeholder,’ which served to provide an overview of the substantial number of statements for the participants. Upon completion of the sorting task, the participants were then asked to label any remaining unlabeled piles. Labelling the remaining piles required them to review the contents of their sorting piles, which in some instances led them to reorganize some or all of the statements or revise some of the labels. Following the sorting task, the participants ranked the statements on a five-point Likert-scale ranging from 1 ‘Not important’ to 5 ‘Very important’. To do so, the first author read all statements aloud in succession. The participants stated their ranking verbally and/or pointed to a visual assessment system. The first author then logged all input into the Group Wisdom computer software.

In the third step, we utilized Group Wisdom for statistical analysis as it is specifically designed for the facilitation of Concept Mapping studies and has been commonly used in previous ID research, e.g., Hanzen et al. [43], Lokman et al. [37], and Vlot-van Anrooij et al. [44]. Through multidimensional scaling, a point map was calculated in which the various statements were plotted based on the number of times these were sorted together by the participants. Those statements sorted together more frequently were presented more closely to each other on the point map. After determining the proximity of the statements, clusters can then be formed on the point map through carrying out a hierarchical cluster analysis. For the resulting cluster map, the relative importance of each cluster toward the central concept (i.e., sexual health for people with mild to borderline ID) was calculated.

In the fourth, and final step, an expert group was organized to interpret the final cluster map during an online meeting, in order to ensure adherence to COVID-19 regulations at that juncture. The expert group consisted of five participants: two experts-by-experience with mild ID, two healthcare psychologists with expertise in sexual support and education of people with mild to borderline ID, and a sexologist experienced in working with people with mild to borderline ID. The expert group was facilitated by two researchers, and video recorded. In preparation for the meeting, the experts received a cluster list, which contained the unlabeled clusters with the respective items. The experts were asked to label the clusters in advance. During the meeting, all clusters were discussed regarding their contents and labelled accordingly. Next, the resulting concept map and labelled clusters were reviewed by the experts for any emerging dimensions.

## Results

**Fig. 1:**
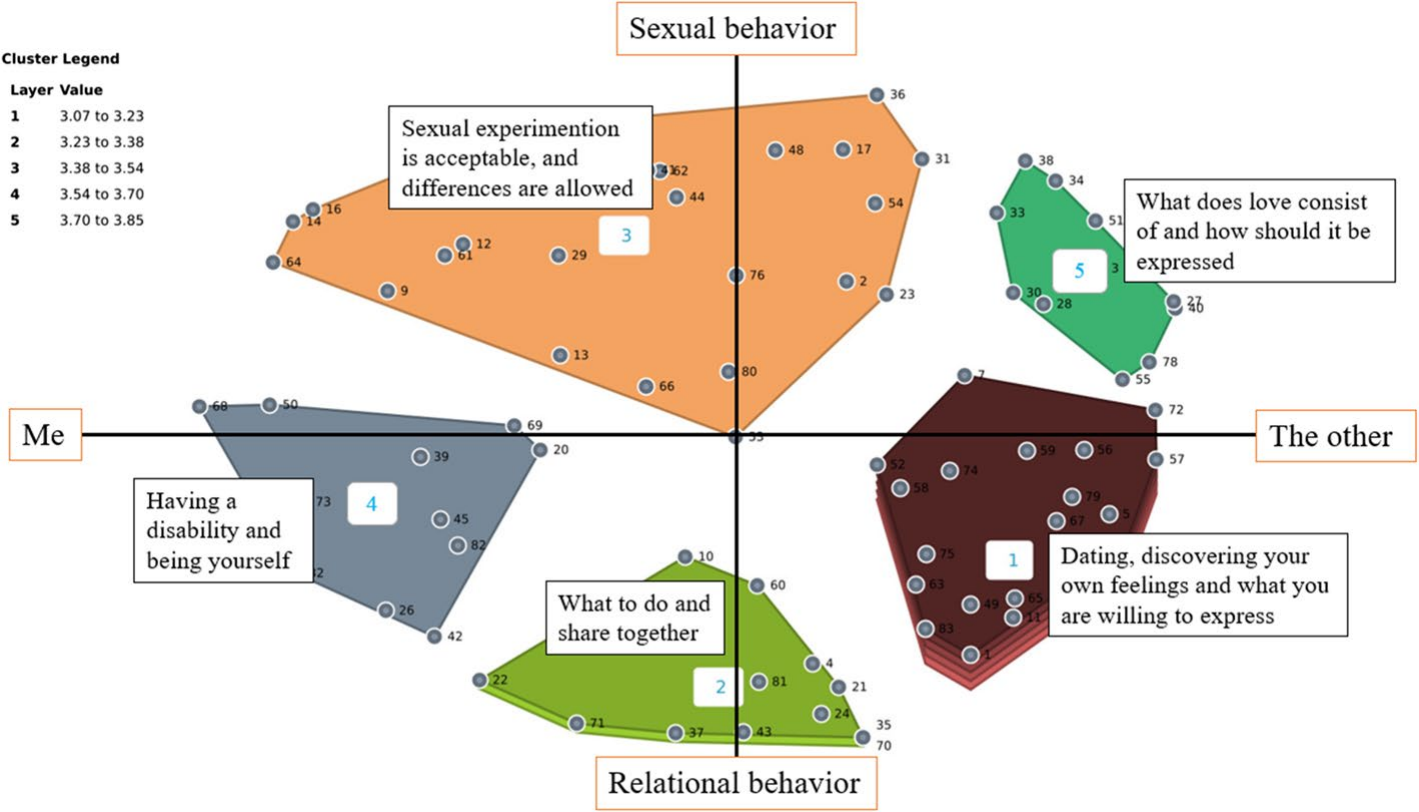
Concept map: the concept of sexual health from the perspective of people with intellectual disabilities. *Note:* more layers indicate that greater importance is placed upon this

In total, 83 statements were gathered that pertained to what people with mild to borderline ID saw sexual health as consisting of an overview of the statements is provided in Online Resource 1. Table 2 provides an overview of the five generated clusters, the number of included statements for each cluster, and their average ratings in the prioritizing task. The clusters are based on how the participants individually prioritized and clustered all the statements; the labels were provided by the expert group. Figure 1 shows how the clusters relate to each other. Below, the clusters of the concept map are further described in descending order of importance.

**Table 2:** Clusters, the number of included statements for each cluster, and their average rating

Cluster number	Label	Number of statements	Average rating
1	Dating, discovering your own feelings and what you are willing to express	21	3.85
2	What to do and share together	14	3.26
3	Sexual experimentation is acceptable, and differences are allowed	25	3.22
4	Having a disability and being yourself	12	3.19
5	What does love consist of and how should it be expressed	11	3.07

According to people with mild to borderline ID, the most important cluster related to sexual health was labelled as ‘Dating, discovering your own feelings and what you are willing to express’ (cluster 1; 21 statements). This cluster described the process of ‘getting to know…’ a possible partner (statement 65) and starting a relationship, which included ‘going on dates’ (statement 1). Personal norms and values concerning dating and starting a relationship were also reported by the participants, such as ‘being open,’ ‘building trust’ and ‘feeling secure’ (statements 67, 75 and 79). Furthermore, setting and knowing one’s boundaries were also deemed to be important for dating and starting a relationship (statement 11).

The second most important cluster with respect to sexual health was labelled: ‘What to do and share together when in a relationship’ (cluster 2; 14 statements), which described behaviors and values associated with relationships. Some people with mild to borderline ID explained that it is important to spend time together in a relationship, such as, for example, ‘going to the movies’ (statement 10), ‘visiting each other at home’ (statement 37) or sharing ‘tea or coffee with a piece of cake’ (statement 70). Furthermore, people with mild to borderline ID reflected on relevant values within a relationship. Alongside ‘daring to be honest’ (statement 43), the statements underscored the importance of being accepted as a person with a disability: ‘I have my disability and I think it is important that I can be myself in a relationship’ (statement 81).

The third important cluster referred to sexual behavior and was labelled: ‘Sexual experimentation is acceptable, and differences are allowed.’ This cluster comprised 25 statements pertaining to sexual health, and included accepting (i.e., allowing) views on different sexual behaviors and sexual orientation. Among these behaviors, people with mild to borderline ID mentioned, among other things, ‘masturbation’ (statement 19), ‘touching each other, physical’ (statement 36), and ‘anal sex’ (statement 41). Besides acceptance, people with mild to borderline ID reported that sexual experimentation provided an opportunity to ‘discover new things together’ (statement 13) and ‘try out stuff together, like using handcuffs and a whip’ (statement 62). The participants found it important for sexual health that both partners take pleasure in sexual experiences (statement 2). Finally, the participants reflected on sexual orientation, stressing that ‘gay and lesbian people are part of our society’ (statement 61) and ‘bisexuality is part and parcel of sexual health’ (statement 12). Through these statements people with ID expressed how individual differences exist and should be allowed.

The fourth important cluster (12 statements) related to sexual health for people with mild to borderline ID was labelled as ‘Having a disability and being yourself’. This cluster included statements pertaining to the meaning of having a mild to borderline ID in a social and sexual environment. In this context, some of the participants expressed that they saw their ID as a barrier to engaging in sexual experiences, and as a source of frustration insofar as they are not able ‘…to conceal their intellectual or physical disability’ (statement 82). Alongside this, other participants shared their experiences of being rejected on the basis of their ID: ‘it is better to share that you have an ID once you know a person for a longer period of time and trust them’ (statement 32).

The cluster that was rated as being least important for sexual health was cluster 5 (11 statements): ‘What does love consist of and how to express it.’ This cluster mostly included behaviors and feelings associated by people with mild to borderline ID with being in love, and how to let a potential partner know that you are in love. For instance, being in love is associated with 'looking at someone lovingly’ (statement 34) and feeling ‘jitters’ (statement 55). In the perspective of sexual health, these feelings of love can be shown to a potential partner through behaviors such as ‘kissing,’ 'winking,’ ‘cuddling,’ and ‘flirting’ (statements 3, 30, 38, and 40).

Further analysis showed how the clusters are distributed over two dimensions, which were interpreted by the expert group. On the first dimension (i.e., the x-axis), the cluster on the left side of the concept map (cluster 4) mostly reflected statements related to sexual selfhood and was labelled by the expert-group as ‘Me’. These statements were opposed by statements on the right side of the cluster map which reflected sexual health in relation to ‘Others’, namely clusters 1 and 5. The second dimension (i.e., the y-axis) reflected behavioral aspects of sexual health, with the cluster on the top of cluster map (i.e., cluster 3) reflecting ‘Sexual behavior’, and the clusters on the bottom of the cluster map reflected ‘Relational behaviors’ (i.e., clusters 1 and 2).

## Discussion

The present study aimed to explore what sexual health consists of according to people with a mild to borderline ID. From the participants’ statements, a group perspective was formed, that identified five clusters: ‘Dating, discovering your own feelings, and what you are willing to express’, ‘What to do and share together’, ‘Sexual experimentation is acceptable, and differences are allowed’, ‘Having a disability and being yourself’, and ‘What does love consist of and how should it be expressed’. The visualization of sexual health illustrates how people with mild to borderline ID referred to characteristics of sexual health, including their sexual selfhood (i.e., how a person perceives their own sexuality) and their sexual socialization (i.e., how a person perceives their sexuality in relation to others). Furthermore, their concept of sexual health included both relational and sexual behavior.

For the people with mild to borderline ID who participated in the present study, the most relevant clusters pertained to relational behavior and sexual socialization. These clusters were prioritized over sexual behavior and included behavioral descriptions (e.g., going on a date and going to the movies together) as well as important personal norms and values for building or having a relationship (e.g., feeling secure, being able to be yourself, and respecting each other’s boundaries). These results are in line with previous research, as they reflect the positive appreciation of romantic relations regarding sexual health [7, 25, 45]. For example, previous research has shown that people with mild to borderline ID pursue a romantic relationship because, among other things, they want to care for somebody, they are seeking company, and they wish to feel accepted [20, 46–48]. Furthermore, previous research also suggests that people with mild to borderline ID prioritize romantic relationships over sexual behavior [49–51]. Prioritizing romantic relationships over sexual behavior might explain, in part, why most people with mild to borderline ID have had romantic relationships, but have had far fewer positive sexual experiences in comparison to people without disabilities [6]. Besides their own prioritization of the statements, people with mild to borderline ID are undoubtedly confronted with multiple barriers with respect to sexual health, including experiencing stigma from support staff and relatives [5, 7]. People with mild to borderline ID who depend on their support staff and relatives for their sexuality-based support and education tend to integrate these negative attitudes from support staff and relatives with their own attitudes [14, 52], which, in turn, potentially leads them to conclude that sexual behaviors are simply not for them. If support staff and relatives were to integrate a positive and holistic perspective on sexual health within their sexuality-based support and education, then it would allow people with mild to borderline ID to make more independent sexual decisions [53–55]. Several promising interventions are being developed [e.g., 56, 57], although the transfer to daily life is still unclear [32, 58]. Further research is needed into exploring the possibility of promoting sexual health through encouraging independent sexual decision-making among people with mild to borderline IDs.

It should be noted, however, that people with mild to borderline ID also prioritize sexual behavior as an important part of their sexual health, in addition to relational behavior. Indeed, the participants in the present study expressed a mainly positive view toward sexual behaviors. These results reflect the positive views toward various forms of sexual behavior found in earlier studies [12]. While previous research identified behaviors like masturbation and petting each other [6, 7], the present study adds anal sex and sexual experimentation. Participants portrayed sexual experimentation as a means through which to develop sexual skills, while, simultaneously, taking care to respect their sexual partner’s boundaries and demanding that their own boundaries also be respected. In this context, sexual experimentation can be regarded as a form of acquiring sexual experience. Therefore, these results are in accordance with previous research on the importance of sexual experiences for developing sexual health [57, 59]. People with mild to borderline ID often require support to be able to engage in sexual experiences, let alone to engage in much-needed sexual experimentation within a safe environment [45]. However, sexuality-based support is too often framed in terms of sexual risk and the prevention of sexual abuse, which serves to restrict rather than encourage safe experimentation [60]. Given that both the present study and prior research demonstrate that people with mild to borderline ID consider mutual boundaries and respect to be important elements of sexual behavior and relationships, there may be no direct need to impose restrictive measures [60]. Instead, focusing on the emotional needs and positive aspects of sexuality is needed to promote sexual health [54, 61]. Based on the results of this study, these needs include creating opportunities for sexual experimentation, while, simultaneously, affirming respectful relationship-behavior.

Another relevant issue in relation to the sexual health of people with mild to borderline ID is their sexual selfhood. In this regard, several participants in the present study appeared to be self-aware of the impact of their disability upon their sexual health, insofar as they believed that potential partners ignored them because of their disability. Consequently, they opted to conceal their disability when dating. For these participants, their sexual selfhood includes an internalized barrier to sexual expression. Previous research has shown how this internalization might stem from both earlier negative experiences with relationships and sexuality [18] and negative experiences with the dominant and often limiting roles of support staff and relatives [14, 62]. Paradoxically, it is only through positive forms of support and education that people with mild to borderline ID can overcome these aforementioned barriers toward their sexual health [4, 58], as has been demonstrated in interventions for example, Kahonde [63], and Van den Toren [64]. Interestingly, despite the significant role of support and education in the promotion of sexual health for people with mild to borderline ID [65], they were not mentioned in the present study. This suggests that people with mild to borderline ID did not consider support and education to be relevant for their sexual health. However, in other recent studies people with mild to borderline ID did consider support and education to be important for their sexual health [7, 12]. One potential reason why support and education went unmentioned in the present study is that some people with mild to borderline ID have learned to avoid sexuality-based support in order to prevent restrictions and punishment from support staff or relatives following their sexual endeavors [27, 66]. However, previous research suggests that people with mild to borderline ID are more receptive to support and education when they have positive experiences with support staff or relatives who match their sexual and educational needs [28]. Hence, for support and sex education to be successful, they must be aligned with the individual needs of people with ID [12, 28]. The fact that people with mild to borderline ID prioritize dating and relationships, as shown in this and other studies [67], indicates that support and education should also prioritize these social dimensions. Further research is thus needed to explore how to develop effective forms of sexual support and education, under which conditions and provided by whom, to better promote sexual health for people with mild to moderate ID as well as to understand their motivation for attending such support and education. Against this background, understanding the concept of sexual health for people with mild to borderline ID from the perspective of support staff and relatives is imperative, and, as such, constitutes an important avenue for future research to focus on.

There are some limitations of the study that must be considered when interpreting the results. First, the study sample was relatively small, consisting of nine participants from a single healthcare organization. Given that the study took place during the COVID-19 pandemic, restrictions on social contact hampered both the sampling and data collection. To adhere to COVID-19 regulations, focus groups were held online, which is considered a valid alternative to face-to-face interviews [68]. Although the research procedure aimed to include participants from various backgrounds to ensure that heterogeneous ideas were taken into account, the results should be interpreted with caution given the local research setting and number of participants. Repeating the research in both other international settings and with a larger number of participants would be valuable. Furthermore, although we did not collect data on gender identity or sexual orientation, these factors might influence their perspectives on sexual health. Therefore, future studies should aim to include a diverse range of participants with respect to sexual orientation and gender identity. Another limitation pertains to the fact that only two women with mild ID agreed to participate in the study. This might reflect the sensitivity of talking about sexuality for women [69], especially with a male researcher. Several preventive actions were undertaken to increase the women’s sense of social safety. Firstly, we organized introductory meetings in which potential participants could have support staff attend with them. Secondly, the online focus groups were run by both a male and female researcher. Finally, the sorting and ranking task was held on a location preferred by the participant, although we explicitly stated that this could not be in their bedroom. After consulting with their support staff, both women agreed to do the task in the office at their housing unit, with support staff in close proximity. Despite these preventive measures, the participants may still have felt unable to speak freely, and thus the results should be interpreted with caution. It is recommended that future research include more women and allow the participants to choose to have a male or female researcher for the sorting and ranking task. Future research should also include an experienced researcher within the expert group to accommodate a research perspective. An additional issue is that the sorting and ranking tasks appeared to be challenging for several participants. To mitigate this, all the participants received practical assistance during these tasks, including reading all statements out loud, completing the task on paper, and prompting the participants to provide labels for the sorting piles at a relatively early stage to help provide them with an overview. Nevertheless, these difficulties may have led the clusters to be less distinctive than desired. Considering the aforementioned limitations, further quantitative research is recomended on a larger scale to confirm the aspects of sexual health and their prioritization, regardless of whether they were identified in this study or not.

In summary, the people with mild to borderline ID in this study described what they believe sexual health consists of. The most relevant clusters pertained to romantic relationships and sexual socialization, followed by sexual behavior and lastly the participants’ sexual selfhood. Various sexual behaviors, including sexual experimentation, were included in their concept of sexual health, along with the desire to respect each other’s boundaries. People with mild to borderline ID demonstrated how their sexual selfhood included their disability, which, in turn, formed a possible barrier in their search for a partner. Further research is needed on support staff and relatives’ concept of sexual health for people with mild to borderline ID, in order to both determine what forms of support and education would best promote sexual health for people with mild to borderline ID and increase the motivation of people with mild to borderline ID to engage with them.

## Supplementary Information

Below is the link to the electronic supplementary material.

Supplementary file1 (PDF 132 KB)

## Data Availability

(Secondary) data and materials used in this research are available.
