# Supplementary material for: Using Concept Mapping to Explore the Perspectives of People with Mild to Borderline Intellectual Disabilities Toward Sexual Health
Source: Sex Disabil. 2023 May 9:1–15. Online ahead of print. doi: 10.1007/s11195-023-09796-w (PMC10169136; doi:10.1007/s11195-023-09796-w)
Supplement: Supplementary file 1 — Supplementary file1 (PDF 132 KB) [file 11195_2023_9796_MOESM1_ESM.pdf]

**Supplementary data**

Data gathered on the focal question: "*Sexual health for people with mild intellectual disabilities consists of …?*" All statements are arranged by clusters.

| **Cluster 1 - Dating, discovering your own feelings and what you are willing to express** | |
|---|---|
| 1 | Going on dates |
| 5 | Making clear agreements |
| 6 | Feeling each other |
| 7 | Giving a rose to a woman |
| 11 | Setting boundaries: what you do want to do and do not want to do |
| 15 | Knowing each other |
| 47 | Taking a stroll together |
| 49 | Finding a partner with good qualities, such as, for example, honesty, spontaneity, reliability, and being yourself and finding it easy to say what you want to say |
| 52 | Setting your own boundaries in what you do not want, such as putting things in your body |
| 56 | Daring to set out on the adventure of a relationship and living a life together |
| 57 | Birth control |
| 58 | Using contraceptives |
| 59 | Sex helps you to learn to trust the other person |
| 63 | Going out on dates to get to know each other |
| 65 | Getting to know each other |
| 67 | Being able to trust each other/feeling secure |
| 72 | Knowing what the other person loves |
| 74 | Not every relationship has to include sex |
| 75 | Being open toward others |
| 79 | Building trust |
| 83 | Knowing other people's boundaries |

| **Cluster 2 - What to do and share together** | |
|---|---|
| 4 | That you know from each other what you are up against |
| 10 | Going to the movies |
| 18 | They need to accept me as I am |
| 21 | First, you need to talk about yourself, what you have been through and that kind of stuff |
| 22 | When you hit it off, you can tell from their character, clean clothes, and clean teeth. |
| 24 | Going to a nightclub |

| | |
|---|---|
| 35 | Buying the other person a drink |
| 37 | Visiting each other at home |
| 43 | Daring to be honest with each other |
| 46 | Everyone should know for themselves what they find pleasurable |
| 60 | Going for a drive together |
| 70 | Having tea or coffee with a piece of cake |
| 71 | Meeting people over 'Facetime' |
| 81 | I have my disability and I think it is important that I can be myself in a relationship |

| **Cluster 3 - Sexual experimentation is acceptable, and differences are allowed** | |
|---|---|
| 2 | It is important that both people find it pleasurable |
| 9 | It is okay to think what you want, but not to do what you want |
| 12 | Bisexuality is part and parcel of sexual health |
| 13 | Daring to discover new things together |
| 14 | Being sexually active on your own |
| 16 | Engaging in sexual activities by yourself can help to remove stress |
| 17 | See if you hit it off |
| 19 | Masturbating |
| 23 | Looking elsewhere if the other person does not talk to you on a dating-app |
| 25 | Do not start talking about sexuality right away, because people might be put off instantly |
| 29 | If one person has an intellectual disability and the other does not, then we are immediately written off |
| 31 | A good relationship should be included in sexual health |
| 36 | Touching each other: being physical with your hands |
| 41 | Trying out anal sex |
| 44 | Being honest about what you do not like sexually |
| 48 | Engaging in sexual behavior allows you to learn about the other person |
| 53 | If you look at each other while trying out things sexually, then you can tell if someone does not like it but does not dare to say that they do not |
| 54 | A girl you like |
| 61 | Gay and lesbian people are a part of our society |
| 62 | Trying out stuff together, like using handcuffs and a whip |
| 64 | Engaging in sexual activity by yourself helps you to deal with things you cannot do |
| 66 | Sex is also possible outside of a relationship, and it is also possible without a relationship |
| 76 | Looking good, not too old, and wrinkly, having a cute face |
| 77 | Engaging in sexual activity by yourself helps you to fall asleep |
| 80 | Above all, be yourself |

| Cluster 4 - Having a disability and being yourself | |
|---|---|
| 8 | Looks are not so important; it is more about the person’s inner self |
| 20 | You can also meet someone and have sex in the woods |
| 26 | There should not be any taboos on what you can discuss together |
| 32 | You can only tell a person later, once you have known them for a longer period, have been on a date and trust them, that you have an intellectual disability |
| 39 | Dating through a dating-app |
| 42 | There are always colleagues who can help you, by making sure that you do not do anything that you should not do |
| 45 | Do not cheat on each other |
| 50 | Sexuality is difficult to talk about |
| 68 | Transgender people are part of sexual health |
| 69 | It is human nature and helps to propagate mankind |
| 73 | The influence from colleagues might be negative |
| 82 | Not everyone can conceal their intellectual or physical disability |

| Cluster 5 - What does love consist of and how should it be expressed | |
|---|---|
| 3 | Kissing |
| 27 | Winking |
| 28 | Going out for dinner |
| 30 | Whistling |
| 33 | Engaging in safe sex together: using condoms and knowing from one another whether you are carrying any diseases |
| 34 | Looking at someone lovingly |
| 38 | Cuddling |
| 40 | Flirting |
| 51 | Starting to fall in love with someone |
| 55 | You get jitters, butterflies in your stomach |
| 78 | Getting engaged |
